# Case Report: Unremitting COVID-19 Pneumonia, Viral Shedding, and Failure to Develop Anti-SARS-CoV-2 Antibodies for More Than 6 Months in Patient with Mantle Cell Lymphoma Treated with Rituximab

**DOI:** 10.4269/ajtmh.21-1010

**Published:** 2022-02-25

**Authors:** Bahadir M. Berktas, Adem Koyuncu

**Affiliations:** Clinic of Chest Diseases, Health Sciences University, Ataturk Chest Diseases and Thoracic Surgery Training and Research Hospital, Ankara, Turkey

## Abstract

Severe cases of COVID-19 are being reported in patients with comorbidities and drug-induced immunosuppression. The risk seems to depend on the type of immunosuppressive agents used, and it is particularly high with rituximab because of its long-lasting effects. We report a 71-year-old man with COVID-19, mantle cell lymphoma, and rituximab-associated immunodeficiency. His COVID-19 clinical course was severe, unremitting, prolonged, and with frequent acute exacerbations requiring hospitalization. Viral shedding and failure to develop anti-severe acute respiratory syndrome coronavirus 2 antibodies continued for at least 6 months.

## INTRODUCTION

COVID-19 has caused more than 4.4 million deaths worldwide. ^1^ Patients with comorbidities and medication- or underlying disease process-induced immunosuppressive conditions including HIV, cancer, and organ transplantation are at greater risk for a severe course of COVID-19. ^2^^,^ ^3^ Differences in the COVID-19 clinical course between immunocompetent and immunosuppressed patients are not well established. ^4^ In drug-induced immunosuppression, the risk seems to depend on the type of immunosuppressive agents used, and is especially high with rituximab, which may be a result of its long-lasting effects, ^5^^,^ ^6^ but more data related to these risks are needed.

Rituximab is an anti-CD20 monoclonal antibody targeting pre-B and mature B cells. ^7^ Rituximab also seems to effect T-cell function, disrupting communication between B cells and T cells, T-cell activation, and other immunomodulatory functions. ^8^ Rituximab is widely used in patients with hematological malignancies and autoimmune disorders, including mantle cell lymphoma, chronic lymphocytic leukemia, immune thrombocytopenia, antineutrophil cytoplasmic antibody-associated vasculitis, rheumatoid arthritis, systemic sclerosis, and systemic lupus erythematosus. ^9^ Rituximab was the top-selling oncology drug, with sales reaching USD $8.58 billion in 2016. Widespread use of rituximab and biosimilars will likely continue. ^10^

Rituximab induces killing of CD20-positive cells directly via complement-mediated cytotoxicity and antibody-dependent cell-mediated cytotoxicity, and indirectly via apoptosis, structural changes, and increasing sensitivity of cancer cell to chemotherapy. ^11^ The half-life of rituximab is long (approximately 3 weeks) and B-cell depletion effects persist for 6 to 12 months. ^12^^,^ ^13^

We describe a case of unremitting COVID-19 with persistent viral shedding in a patient with lymphoma and rituximab-associated immunodeficiency who experienced clinical worsening late in his clinical course. Moreover, he demonstrated ongoing replication of infectious severe acute respiratory syndrome coronavirus 2 (SARS-CoV-2) for at least 6 months.

## CASE PRESENTATION

A 71-year-old man presented to our hospital on October 21, 2020 with 4 days of cough. A nasopharyngeal swab was collected, and he was diagnosed with COVID-19 via reverse transcription–polymerase chain reaction (RT-PCR).

His medical history was significant for mantle cell lymphoma, diagnosed in 2013, which was immunohistochemically positive for CD20, CD5, BCL2, and cyclin D1. He was treated initially with rituximab; cyclophosphamide, doxorubicin, vincristine, and prednisone therapy; and autologous bone marrow transplantation.

On disease recurrence in 2018, the patient was treated with salvage therapy of rituximab plus bendamustine for six cycles, then was continued on maintenance therapy with rituximab dosed every 3 months. His last dose of rituximab was delayed because of COVID-19-related lockdowns and travel restrictions. He was diagnosed with COVID-19 4 months after receiving his last dose.

**Figure 1.**
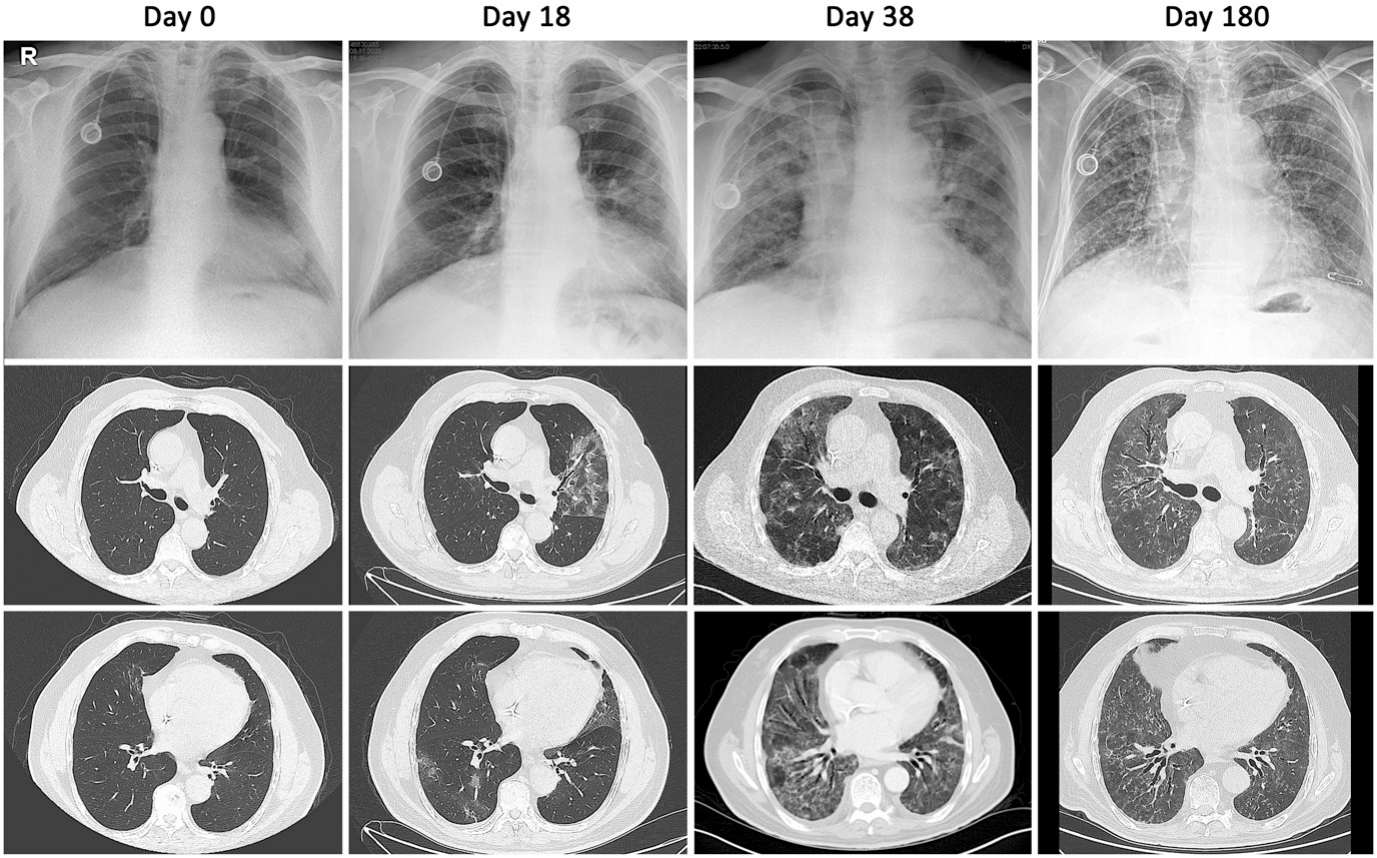
Chest X-rays and computed tomographic scans across the disease course. Days are given since the first positive reverse transcription–polymerase chain reaction results. Computed tomography showed a small area of ground-glass opacity in the left upper lobe on day 0. Progressive ground-glass opacities more prominent at the left upper and right lower lobes indicate prolonged evolution of COVID-19 pneumonia on day 18. Bilaterally disseminated ground-glass opacities and crazed paving pattern with areas of consolidations were noted on day 38. Fibrotic-like changes were seen on day 180.

On presentation, the patient’s vital signs were stable, including 98% oxygen saturation on room air. Physical examination, chest radiograph, and laboratory findings were unremarkable. Computed tomography (CT) of the chest showed only a small area of ground-glass opacity in the left upper lobe (Figure 1). Despite mild symptoms, the patient was hospitalized for clinical monitoring because of his risk profile. He was treated with favipiravir for 5 days in accordance with our national COVID-19 treatment guide, low-molecular weight heparin for antithrombotic prophylaxis related to COVID-19, ^14^ and was discharged to home quarantine on day 7.

After 18 days, the patient returned to the emergency department with fever and dyspnea. His vital signs were as follows: temperature, 38.2°C; heart rate, 102 beats/min; blood pressure, 130/86 mmHg; and oxygen saturation, 96% on 2 L/min oxygen therapy by nasal canula. C-reactive protein (CRP) was elevated at 33.4 mg/L, and ferritin at 527 ng/mL. His lymphocyte count was depleted to 710 lymphocytes/µL. Chest CT showed progressive, bilateral ground-glass opacities predominantly in the left upper lobe, although the patient still had only minimal respiratory symptoms (Figure 1). We commenced treatment again with favipiravir, as well as dexamethasone 6 mg/day and cefoperazone with sulbactam for probable bacterial coinfection.

After 5 days of continued fever, the patient’s antibiotics were changed to meropenem plus linezolid because of suspected hospital-acquired infection. CRP was elevated at 64.2 mg/L; ferritin, at 1,650 ng/mL. Blood cultures showed no bacterial growth. COVID-19 was thought to be the cause of fever, so remdesivir and pulse corticosteroid therapy (120 mg/day prednisolone) were started (day 23). The fever subsequently improved to less than 38°C, and CRP and ferritin levels decreased slightly. RT-PCR examination for SARS-CoV-2 was negative.

Unfortunately, the patient’s fever recurred 2 days later. A chest X-ray showed that the pneumonia had gradually worsened, and laboratory results revealed that his inflammatory markers were again extremely elevated. Treatment with anakinra, an interleukin 1 antagonist, was initiated, which further depleted his lymphocyte count to 200 lymphocytes/µL. Repeat RT-PCR for SARS-CoV-2 was again positive. Antibodies to SARS-CoV-2 were not detected on serological testing with EUROIMMUN anti-SARS-CoV-2 IgG ELISA. Rituximab-related B-cell depletion was thought to be the cause of lymphocytopenia, so 2 U of convalescent plasma were transfused. Despite these treatments, the patient’s fever continued and his oxygen requirement increased to a high-flow nasal cannula (HFNC) at 50 L/min, so he was transferred to the intensive care unit (ICU) on day 38. Repeat CT revealed bilaterally disseminated ground-glass opacities and a crazed paving pattern with areas of consolidation (Figure 1).

**Figure 2.**
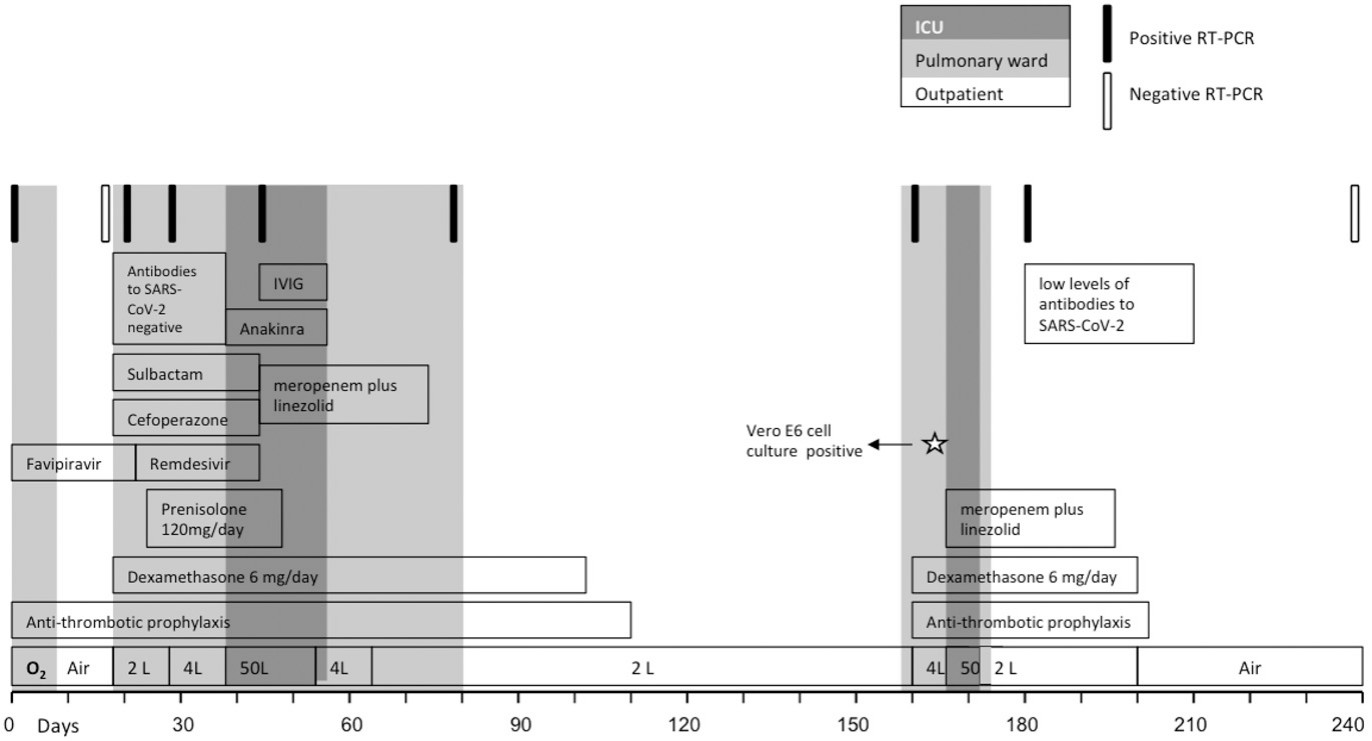
Timeline of disease course, treatments, severe acute respiratory syndrome coronavirus 2 (SARS-CoV-2) reverse transcription–polymerase chain reaction (RT-PCR) tests, antibody tests, and Vero E6 cell line culture from day 0 to day 240. ICU = intensive care unit; IVIG = intravenous immunoglobulin; O_2_ = oxygen.

The patient was maintained on noninvasive mechanical ventilation and HFNC interchangeably. His fever continued intermittently, but blood, sputum, and deep tracheal aspiration cultures; *Aspergillus*; *Pneumocystis jirovecii*; and *cytomegalovirus* PCR were all negative. Repeat SARS-CoV-2 RT-PCR showed persistent positive results (Figure 2). His IgG IgM levels decreased to 627 mg/dL and 31.1 mg/dL, respectively. Intravenous immunoglobulin (IVIG) therapy was initiated in the ICU as an adjunctive drug for possible attenuation of inflammatory responses. ^15^ His oxygen needs decreased gradually and his febrile periods shortened. He was transferred to the pulmonology ward on day 57 and discharged on day 80. His RT-PCR test was still positive for SARS-CoV-2 on day 80 (Figure 2).

The patient was later admitted to the rehabilitation clinic for continued oxygen support and dyspnea symptoms 78 days later (day 158), at which time he continued to test positive for SARS-CoV-2 on RT-PCR. His SARS-CoV-2-positive sample was then cultured in a Vero E6 cell line and inspected for cytopathic effect. ^16^ A SARS-CoV-2-induced cytopathic effect was observed, and culture supernatant was confirmed by PCR (Figure 2). Unfortunately, we could not perform genotyping or sequencing during his disease course.

Eight days later, on day 166, the patient was transferred to the ICU for new-onset fever, rapidly increasing dyspnea, and increased oxygen support needs. Antibiotic treatment with meropenem plus linezolid, and HFNC therapy was commenced. Despite the lack of bacterial growth on blood culture, his respiratory condition improved quickly after these treatments. He was discharged with home oxygen support on day 172.

At follow-up on day 180, chest CT showed bilateral fibrotic-like changes (Figure 1), and, for the first time, serological testing detected low levels of antibodies to SARS-CoV-2 (IgG, 3.60; range, 0–0.99). Clinically, the patient continued to have symptoms of dyspnea on exertion, and fatigue, and still used home oxygen therapy (2 L/min at night). On day 240, an RT-PCR test for SARS-CoV-2 was negative. His mantle cell lymphoma is currently in remission.

## DISCUSSION

Rituximab causes rapid, profound, and long-lasting (6–12 months) B-cell depletion, resulting in a well-established risk of infection. ^13^ B-cell repopulation is delayed by a mean time of 8 months after treatment in patients with rheumatoid arthritis. ^17^ One patient with systemic lupus erythematosus remained B-cell depleted for more than 4 years. ^18^ Some T cells and natural killer (NK) cells also express low levels of CD20; therefore, T-cell and NK-cell depletion during rituximab treatment has been demonstrated, with repopulation after a mean of 5 months. ^17^ Depletion of T-helper lymphocytes as seen in HIV-infected patients ^19^ may also contribute to risk of infection.

In our patient, a SARS-CoV-2 infection occurred 4 months after his last rituximab dose, which is within the expected time frame of B-cell, T-cell, and NK-cell depletion, which placed him in an immunosuppressive state.

Rapid exacerbation of pneumonia occurs usually 1 to 2 weeks after the first symptoms appear during the general course of COVID-19. ^20^ In our patient, it started after 3 weeks and worsened after 4 weeks. This delayed occurrence of pneumonia has been reported frequently in COVID-19 patients treated with rituximab. ^21^^–^ ^23^ It is posited that rituximab might initially, but insufficiently, limit the cytokine storm, leading to a delayed worsening of clinical presentation. ^23^ This delayed response is comparable to that seen in immune reconstitution inflammatory syndrome, in which the immune response to pathogens becomes apparent as the immune system is restored. ^4^ Despite the delayed worsening of symptoms, outcomes can be remarkably poor, including respiratory failure, thrombotic complications, and death. ^9^^,^ ^24^ Contrary to initial suggestions that immunosuppressive states such as HIV‐related immunosuppression may have mortality benefits, greater COVID-19-related case fatality rates are demonstrated in patients with HIV, cancer, and drug-induced immunosuppression. ^25^

Most COVID-19 patients demonstrate seroconversion 7 to 14 days after infection, which often coincides with symptom resolution. ^20^ The COVID-19 clinical course in our patient was severe, unremitting, prolonged, and with frequent acute exacerbations that required hospitalization. His mantle cell lymphoma and rituximab-associated immunosuppression are likely the reasons for his protracted clinical course. He was treated with antivirals, broad-spectrum antibiotics, corticosteroids, oxygen support, anakinra, convalescent plasma, and IVIG. Some reports in the literature describe the successful use of convalescent plasma and IVIG in COVID-19 patients with a history of rituximab treatment, ^26^^,^ ^27^ but we observed only partial improvement with these treatments.

Repeat SARS-CoV-2 RT-PCR testing showed persistent positive results for at least 6 months in our patient. This is consistent with previous reports that have noted that immunosuppressed patients shed the active replicating virus over a longer period of time than immunocompetent patients. ^24^ The persisting SARS-CoV-2 viremia and recurring fevers reported after rituximab therapy may thus be related to suppressed B-cell function. ^28^

Our patient did not develop anti-SARS-CoV-2 antibodies for 6 months, which is consistent with the impaired humoral response frequently observed with rituximub. ^23^ It may be that B-cell depletion contributed to his prolonged lymphocytopenia and inability to produce antibodies. Cases such as this emphasize the challenges in managing immunocompromised patients, who are persistent shedders and sources of transmission. These patients may be reservoirs of antigenically novel viruses, ^29^ and may experience decreased efficacy of the SARS-CoV-2 vaccine. ^30^

In conclusion, patients with rituximab-related immunodeficiency might differ in their degree of viral shedding, immune clearance kinetics, and severity of COVID-19 disease. Therefore, patient education related to COVID-19 precautions is important after rituximab application. Effectively managing COVID-19 in immunosuppressed patients who are shedding virus persistently is imperative for preventing new variants of SARS-CoV-2.

## References

[1] Office for the Coordination of Humanitarian Affairs Services, reliefweb , 2021. *Coronavirus Disease (COVID-19): Weekly Epidemiological Update (24 August 2021).* Available at: https://reliefweb.int/report/world/coronavirus-disease-covid-19-weekly-epidemiological-update-24-august-2021. Accessed February 11, 2022.

[2] He W 2020. COVID-19 in persons with haematological cancers. Leukemia 4: 1637–1645. 10.1038/s41375-020-0836-7 PMC7180672 32332856

[3] Mohammed AH Blebil A Dujaili J Rasool-Hassan BA , 2020. The risk and impact of COVID-19 pandemic on immunosuppressed patients: cancer, HIV, and solid organ transplant recipients. AIDS Rev 22: 151–157. 33118527 10.24875/AIDSRev.20000052

[4] Otsuka Y Kobayashi T , 2020. Case report: a patient with COVID-19 under myelosuppression induced by chemotherapy. Am J Trop Med Hyg 103: 1983–1985. 32940203 10.4269/ajtmh.20-0678 PMC7646807

[5] Strangfeld A 2021. Factors associated with COVID-19-related death in people with rheumatic diseases: results from the COVID-19 Global Rheumatology Alliance physician-reported registry. Ann Rheum Dis 80: 930–942. 33504483 10.1136/annrheumdis-2020-219498 PMC7843211

[6] Vasconcelos J Portugal R Torres R Falcao S , 2021. Intravenous immunoglobulin as a therapeutic option for patients with worsening COVID-19 under rituximab. BMJ Case Rep 14: e243338. 10.1136/bcr-2021-243338 PMC8240564 34183316

[7] Kessel A Rosner I Toubi E , 2008. Rituximab: beyond simple B cell depletion. Clin Rev Allergy Immunol 34: 74–79. 18240027 10.1007/s12016-008-8074-1

[8] Tudesq J-J 2018. Clinical and microbiological characteristics of the infections in patients treated with rituximab for autoimmune and/or malignant hematological disorders. Autoimmun Rev 17: 115–124. 29180125 10.1016/j.autrev.2017.11.015

[9] Hoffmann MS Siddhartha G , 2021. Delayed COVID-19 respiratory failure in patients with lymphoma on rituximab-based chemoimmunotherapy. Clin Lymphoma Myeloma Leuk 21: e548–e550. 33712408 10.1016/j.clml.2021.02.009 PMC7910130

[10] Pierpont TM Limper CB Richards KL , 2018. Past, present, and future of rituximab: the world’s first oncology monoclonal antibody therapy. Front Oncol 8: 163. 29915719 10.3389/fonc.2018.00163 PMC5994406

[11] Cerny T 2002. Mechanism of action of rituximab. Anticancer Drugs 13: *(Suppl 2)*: S3–S10. 10.1097/00001813-200211002-00002 12710585

[12] Regazzi MB 2005. Pharmacokinetic behavior of rituximab: a study of different schedules of administration for heterogeneous clinical settings. Ther Drug Monit 27: 785–792. 16306856 10.1097/01.ftd.0000184162.60197.c1

[13] Marco H 2014. The effect of rituximab therapy on immunoglobulin levels in patients with multisystem autoimmune disease. BMC Musculoskelet Disord 15: 178. 24884562 10.1186/1471-2474-15-178 PMC4038057

[14] Di Perri G , 2020. The rationale for low-molecular weight heparin (LMWH) use in SARS-CoV-2 infection. Infez Med 28: 52–56. 32532939

[15] Tzilas V Manali E Papiris S Bouros D , 2020. Intravenous immunoglobulin for the treatment of COVID-19: a promising tool. Respiration 99: 1087–1089. 33212437 10.1159/000512727 PMC7801989

[16] Matsuyama S 2020. Enhanced isolation of SARS-CoV-2 by TMPRSS2-expressing cells. Proc Natl Acad Sci USA 177: 7001–7003. 10.1073/pnas.2002589117 PMC7132130 32165541

[17] Leandro MJ Cambridge G Ehrenstein MR Edwards JC , 2006. Reconstitution of peripheral blood B cells after depletion with rituximab in patients with RA. Arthritis Rheum 54: 613–620. 16447239 10.1002/art.21617

[18] Leandro MJ Cambridge G Edwards JC Ehrenstein MR Isenberg DA , 2005. B-cell depletion in the treatment of patients with systemic lupus erythematosus: a longitudinal analysis of 24 patients. Rheumatology 44: 1542–1545. 16188950 10.1093/rheumatology/kei080

[19] Bagby GJ 2015. Alcohol and HIV effects on the immune system. Alcohol Res 37: 287–297. 26695751 10.35946/arcr.v37.2.12 PMC4590624

[20] Huang C 2020. Clinical features of patients infected with 2019 novel coronavirus in Wuhan, China. Lancet 395: 497–506. 31986264 10.1016/S0140-6736(20)30183-5 PMC7159299

[21] Leipe J Wilke EL Ebert MP Teufel A Reindl W , 2020. Long, relapsing, and atypical symptomatic course of COVID-19 in a B-cell-depleted patient after rituximab. Semin Arthritis Rheum 50: 1087–1088. 32916559 10.1016/j.semarthrit.2020.06.013 PMC7833880

[b22] Guilpain P 2021. Rituximab for granulomatosis with polyangiitis in the pandemic of COVID-19: lessons from a case with severe pneumonia. Ann Rheum Dis 80: e10. 32312768 10.1136/annrheumdis-2020-217549

[23] Avouac J Airó P Carlier N Matucci-Cerinic M Allanore Y , 2021. Severe COVID-19-associated pneumonia in 3 patients with systemic sclerosis treated with rituximab. Ann Rheum Dis 80: e37. 10.1136/annrheumdis-2020-217864 32503849

[24] Aydillo T 2020. Shedding of viable SARS-CoV-2 after immunosuppressive therapy for cancer. N Engl J Med 383: 2586–2588. 33259154 10.1056/NEJMc2031670 PMC7722690

[25] Kanwugu ON Adadi P , 2021. HIV/SARS-CoV-2 coinfection: a global perspective. J Med Virol 93: 726–732. 32692406 10.1002/jmv.26321 PMC7404432

[26] Wright Z Bersabe A Eden R Bradley J Cap A , 2021. Successful use of COVID-19 convalescent plasma in a patient recently treated for follicular lymphoma. Clin Lymphoma Myeloma Leuk 21: 66–68. 32682684 10.1016/j.clml.2020.06.012 PMC7315985

[27] Vasconcelos J Portugal R Torres R , 2021. Intravenous immunoglobulin as a therapeutic option for patients with worsening COVID-19 under rituximab. BMJ Case Rep 14: e243338. 10.1136/bcr-2021-243338 PMC8240564 34183316

[28] Tepasse PR 2020. Persisting SARS-CoV-2 viremia after rituximab therapy: two cases with fatal outcome and a review of literature. Br J Haematol 190: 185–188. 32557623 10.1111/bjh.16896 PMC7300950

[29] Hoon Baang J 2021. Prolonged severe acute respiratory syndrome coronavirus 2 replication in an immunocompromised patient. J Infect Dis 223: 23–27. 33089317 10.1093/infdis/jiaa666 PMC7797758

[30] Houot R , 2020. Could anti-CD20 therapy jeopardise the efficacy of a SARS-CoV-2 vaccine? Eur J Cancer 136: 4–6. 32619884 10.1016/j.ejca.2020.06.017 PMC7315961

